# Distinct therapeutic profiles of ketamine in treatment-resistant depression: an exploratory analysis

**DOI:** 10.1093/ijnp/pyag021

**Published:** 2026-04-30

**Authors:** Kuan-I Liu, Wei-Chen Lin, Cheng-Ta Li, Ya-Mei Bai, Tung-Ping Su, Mu-Hong Chen

**Affiliations:** Department of Psychiatry, Taipei Veterans General Hospital, Taipei, Taiwan; Department of Psychiatry, Taipei Veterans General Hospital, Taipei, Taiwan; Division of Psychiatry, Faculty of Medicine, National Yang Ming Chiao Tung University, Taipei, Taiwan; Institute of Brain Science, College of Medicine, National Yang Ming Chiao Tung University, Taipei, Taiwan; Department of Psychiatry, Taipei Veterans General Hospital, Taipei, Taiwan; Division of Psychiatry, Faculty of Medicine, National Yang Ming Chiao Tung University, Taipei, Taiwan; Institute of Brain Science, College of Medicine, National Yang Ming Chiao Tung University, Taipei, Taiwan; Department of Psychiatry, Taipei Veterans General Hospital, Taipei, Taiwan; Division of Psychiatry, Faculty of Medicine, National Yang Ming Chiao Tung University, Taipei, Taiwan; Institute of Brain Science, College of Medicine, National Yang Ming Chiao Tung University, Taipei, Taiwan; Department of Psychiatry, Taipei Veterans General Hospital, Taipei, Taiwan; Division of Psychiatry, Faculty of Medicine, National Yang Ming Chiao Tung University, Taipei, Taiwan; Institute of Brain Science, College of Medicine, National Yang Ming Chiao Tung University, Taipei, Taiwan; Department of Psychiatry, Cheng Hsin General Hospital, Taipei, Taiwan; Department of Psychiatry, Taipei Veterans General Hospital, Taipei, Taiwan; Division of Psychiatry, Faculty of Medicine, National Yang Ming Chiao Tung University, Taipei, Taiwan; Institute of Brain Science, College of Medicine, National Yang Ming Chiao Tung University, Taipei, Taiwan

**Keywords:** ketamine, treatment-resistant depression, suicide, Maudsley staging method, precision medicine

## Abstract

**Objective:**

Intravenous ketamine demonstrates rapid efficacy for treatment-resistant depression (TRD). However, its impact on individual depressive symptoms and whether the degree of treatment resistance moderates these effects remain unclear. This study aimed to dissect symptom-specific trajectories and examine the moderating role of the degree of treatment resistance.

**Methods:**

We conducted a post-hoc analysis of pooled data from two randomized, double-blind, controlled trials involving 154 adults with TRD receiving subanesthetic ketamine or control. Depressive symptoms were assessed using the Montgomery-Åsberg Depression Rating Scale (MADRS) over 15 days. Linear mixed-effect models (LMM) analyzed total scores, while generalized estimating equations (GEE) assessed item-level improvement. The Maudsley Staging Method (MSM) was used to quantify the degree of treatment resistance and evaluate its moderating effect.

**Results:**

Ketamine elicited a significant reduction in MADRS total scores (*P* < .001). Item-level analysis revealed significant improvements in seven symptoms, including apparent sadness, inner tension, and suicidal thoughts (*P* < .05). Crucially, a significant treatment × MSM interaction was found for apparent sadness (*P* = .002) and inner tension (*P* = .024), indicating attenuated efficacy in patients with higher resistance. Conversely, anti-suicidal efficacy was robust and not significantly moderated by resistance severity (*P* = .083).

**Conclusions:**

Ketamine demonstrates broad efficacy in TRD but may exhibit symptom-specific divergence: anti-suicidal effects appear robust across resistance levels, whereas improvements in apparent sadness and inner tension may be attenuated by greater resistance severity. These exploratory findings warrant confirmation in prospective studies.

**Trial registration:**

UMIN Clinical Trials Registry (UMIN-CTR): Registration number: UMIN000016985 (registered June 30, 2016), UMIN000023581 (registered December 31, 2018), and UMIN000033916 (registered November 30, 2021).

**Significant outcomes**

1) Intravenous ketamine showed rapid anti-suicidal efficacy in patients with treatment-resistant depression (TRD), which was not significantly moderated by the baseline severity of treatment resistance.
2) Improvement in apparent sadness and inner tension was significantly moderated by the degree of treatment resistance, with attenuated efficacy observed in patients with higher Maudsley Staging Method (MSM) scores.
3) These exploratory findings raise the hypothesis that a symptom-specific stratified therapeutic approach may be warranted. Future research should examine whether ketamine could serve as an acute intervention for suicidal crises across the resistance spectrum, and whether improvements in specific affective symptoms may require augmentation strategies in highly refractory cases.

**Limitations**

1) Limited generalizability: The study population consisted exclusively of Han Chinese patients from a single medical center, which may restrict the applicability of these symptom-specific findings to other ethnic groups or cultural contexts, given potential genetic and cultural variations.
2) Methodological constraints: This was a post-hoc analysis of pooled data with a heterogeneous control group (saline or midazolam). Functional unblinding may have occurred in both treatment directions, and formal blinding assessment was not conducted in either contributing trial. Sensitivity analyses adjusting for study source confirmed the robustness of all primary findings.
3) Study design duration: The protocol involved a single infusion with a short 15-day follow-up period, precluding conclusions regarding the long-term durability of the observed symptom divergence or the efficacy of repeated dosing regimens.

## Graphical Abstract

**Graphical Abstract:**
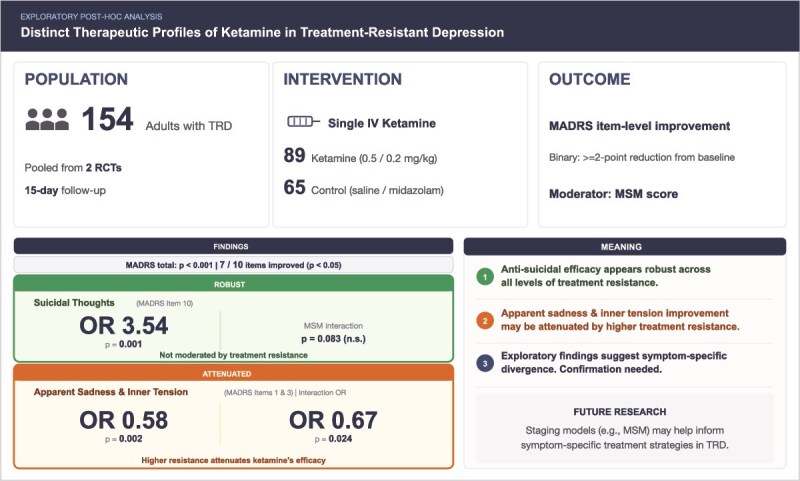

## Introduction

Depression stands as a steadily rising cause of global disability, imposing escalating socioeconomic costs and heightened expenditures on healthcare systems.^1^ Despite the availability of standard pharmacological interventions, approximately one-third of patients fail to derive clinical benefit from at least two adequate medication trials, a condition clinically defined as treatment-resistant depression (TRD).^2^ Compared to responsive major depressive disorder, this difficult-to-treat subpopulation faces a significantly heavier burden, characterized by diminished quality of life, disproportionate healthcare expenditures, and frequent relapse.^3^^,^^4^ Most critically, TRD is linked to substantially elevated rates of all-cause mortality and suicide, highlighting an urgent necessity for more effective, rapid-acting therapeutic strategies.^2^^,^^5^

Historically, therapeutic strategies for TRD were confined to monoaminergic modulation, which frequently offered inadequate symptom resolution.^6^ The discovery of ketamine’s rapid antidepressant properties marked a pivotal advance in psychiatric care,^7^ although the precise mechanisms underlying its efficacy remain debated, with emerging evidence implicating opioid receptor signaling in addition to N-methyl-D-aspartate (NMDA) receptor antagonism.^8–11^ Although intranasal esketamine has secured regulatory approval, intravenous (IV) racemic ketamine at standard doses (eg, 0.5 mg/kg) endures as the research gold standard, distinguished by its complete bioavailability.^12^ Accumulating evidence, notably from trials within Taiwanese cohorts, demonstrates that optimal subanesthetic infusion can yield rapid and robust alleviation of both depressive symptoms and suicidal ideation, offering a vital therapeutic option for patients refractory to conventional regimens.^12^^,^^13^

Antidepressant efficacy is conventionally established by reducing aggregate scores on standardized instruments, such as the Montgomery-Åsberg Depression Rating Scale (MADRS).^14^^,^^15^ While these composite indices serve as valuable benchmarks for overall severity, they inevitably conflate diverse symptoms into a singular metric, potentially obscuring specific therapeutic patterns.^16^ Depression is inherently heterogeneous, comprising a constellation of emotional, cognitive, and vegetative manifestations that may not respond uniformly to pharmacological intervention.^17^ Consequently, relying solely on total scores may mask partial improvements or obscure the specific symptom clusters driving therapeutic benefit.

Just as symptom presentation is inherently heterogeneous, the characterization of treatment resistance also extends beyond a simple binary classification. The Maudsley Staging Method (MSM) offers a multidimensional framework to quantify the severity of resistance, integrating clinical variables such as illness duration and symptom intensity into a continuous staging index.^18^ While higher resistance typically predicts poorer outcomes in conventional antidepressant trials, and emerging evidence suggests a similar attenuation of overall response to ketamine,^19^ it remains unclear whether this pattern applies uniformly across distinct symptom domains. Specifically, establishing whether the impact of treatment resistance varies across individual depressive symptoms represents a significant gap in current precision psychiatry literature.^20^

To address these knowledge gaps, the present study conducted a post-hoc analysis of data derived from randomized, double-blind, controlled trials involving patients with TRD receiving subanesthetic doses of intravenous ketamine. Our study pursued a two-tiered objective: verifying the overall antidepressant efficacy of ketamine using aggregate MADRS scores, while concurrently dissecting specific symptom trajectories to identify the individual symptoms driving this improvement. Furthermore, in an exploratory analysis, we investigated whether the severity of treatment resistance, as quantified by the MSM, moderates the therapeutic response of these individual items. By doing so, we sought to determine if the degree of resistance differentially influences the resolution of specific depressive symptoms.

## Materials and methods

### Study design and participants

This study was conducted as a post-hoc analysis of pooled data derived from two randomized, double-blind, controlled clinical trials performed at Taipei Veterans General Hospital and at Cheng Hsin General Hospital.^12^^,^^13^ A third trial (reference ^16^) was considered but excluded from the analysis, as its follow-up schedule did not include a post-infusion Day-15 MADRS assessment; its characteristics are described in Supplementary Table S1.^21^ We enrolled adult patients (aged 20–65 years) who met the criteria for major depressive disorder (MDD) or bipolar disorder according to the Diagnostic and Statistical Manual of Mental Disorders, fifth edition and were currently experiencing a major depressive episode. All participants fulfilled the definition of TRD, characterized by a lack of response to at least two adequate trials of antidepressants or mood stabilizers.^2^^,^^22^ Exclusion criteria consistently applied across the three trials included a history of alcohol or substance use disorders (including abuse or dependence, excluding nicotine) and major medical or neurological illnesses (eg, stroke or seizure) that could compromise safety during the infusion procedure. Participants were stratified into a ketamine group and a control group based on their original trial assignment; notably, given the pooled nature of this analysis, the two groups were not specifically matched for baseline demographic or clinical characteristics.

### Clinical assessments and procedures

Participants received a single intravenous infusion of ketamine (0.5 or 0.2 mg/kg) or a control agent (normal saline or midazolam) administered over 40 minutes. Depressive symptoms were assessed using the MADRS at baseline and sequentially on Days 2, 3, 5, 7, and 15 post-infusion. Previous research supports the utility of MADRS assessments at such frequent intervals to characterize the rapid onset of efficacy in treatment-resistant depression.^14^^,^^23^ The MADRS is a 10-item clinician-rated scale (total score 0–60) comprising ten distinct symptom domains: Apparent sadness (Item 1), Reported sadness (Item 2), Inner tension (Item 3), Reduced sleep (Item 4), Reduced appetite (Item 5), Concentration difficulties (Item 6), Lassitude (Item 7), Inability to feel (Item 8), Pessimistic thoughts (Item 9), and Suicidal thoughts (Item 10).^14^

To quantify the multidimensional severity of treatment resistance, the MSM was administered at baseline. This clinician-rated instrument provides a composite score derived from three key prognostic dimensions: the duration of the current depressive episode, baseline symptom severity, and the total number of failed adequate treatment trials. Higher cumulative scores on the MSM indicate a greater degree of therapeutic resistance.^18^ Additionally, baseline demographic data, specifically age and sex, were recorded to serve as covariates for statistical adjustment.

### Statistical analysis

Data analyses were performed using R version 4.5.2 (R Foundation for Statistical Computing). Baseline demographic and clinical characteristics were summarized using descriptive statistics, with continuous variables presented as mean and standard deviation and categorical variables as frequencies and percentages.

For the primary outcome, the longitudinal trajectory of depressive symptom severity as measured by the MADRS total score was analyzed using linear mixed-effect models (LMM). This approach enabled the inclusion of all available data points across multiple follow-up visits, specifically Days 2, 3, 5, 7, and 15. The model incorporated treatment group, time, and the group x time interaction as fixed effects, with the unstructured covariance matrix employed to account for within-subject correlations. To control for potential baseline imbalances in this pooled cohort, age and sex were included as covariates in the model. Estimated coefficients and their corresponding 95% CIs were calculated.

As an exploratory item-level analysis to dissect the specific symptom domains responsive to treatment, we utilized generalized estimating equations (GEE) based on logistic regression models. Unlike the continuous aggregate total score, individual item scores are ordinal and skewed; consequently, this binary framework was adopted to ensure statistical robustness and to estimate the probability of clinically meaningful improvement. Based on the established minimal important difference (MID) for the MADRS, item-level improvement was specifically defined as a reduction of at least 2 points from baseline.^24^ We estimated the odds ratios (ORs) and 95% CIs for symptom improvement on each of the 10 MADRS items in the ketamine group relative to the control group. An autoregressive correlation structure was specified to model the dependency of repeated measures over time, and models were adjusted for age and sex.

Finally, a focused exploratory analysis was conducted specifically on the individual depressive symptom items that demonstrated a statistically significant treatment effect in the exploratory item-level analysis. In this step, the MSM total score was introduced into the GEE models as a continuous moderator. We specifically examined the interaction term between treatment and MSM to determine if the magnitude of benefit for specific symptoms was contingent upon the patient’s baseline level of treatment resistance. All statistical tests were two-tailed, with a significance threshold set at a *P*-value less than .05. To assess robustness against between-trial heterogeneity, a sensitivity analysis was conducted incorporating study source as a fixed covariate in all GEE models^12^^,^^13^; results are presented in Supplementary Tables S2 and S3.

## Results

### Demographic And Clinical Characteristics

A total of 154 participants with TRD were included in the final analysis, comprising 89 patients in the ketamine group and 65 in the control group. The overall mean age of the study population was 41.0 years (SD = 13.23), and the majority of participants were female (72.1%). The mean duration of illness was 11.4 years (SD = 8.71), with an average age of onset at 29.9 years (SD = 12.0). Regarding diagnostic categories, 89.0% of patients were diagnosed with Major Depressive Disorder, while 3.2% and 7.8% were diagnosed with Bipolar I and Bipolar II Disorder, respectively. Mean baseline MADRS total scores were 35.1 (SD = 5.95) in the ketamine group and 37.1 (SD = 4.52) in the control group. Regarding treatment resistance severity, the mean MSM total score was 9.2 (SD = 1.92) in the ketamine group and 9.7 (SD = 1.83) in the control group. Demographic and clinical characteristics are summarized in Table 1.

**Table 1:** Baseline demographic and clinical characteristics of all patients with treatment-resistant depression.

Variable	Ketamine group (*n* = 89)	Control group (*n* = 65)	Overall (*n* = 154)
Age (years)			
Mean (SD)	40.8 (13.91)	41.1 (12.35)	41.0 (13.23)
Duration of illness (years)			
Mean (SD)	11.1 (8.77)	11.9 (8.66)	11.4 (8.71)
Diagnosis, *n* (%)			
Major depressive disorder	80 (89.9)	57 (87.7)	137 (89.0)
Bipolar I disorder	3 (3.3)	2 (3.1)	5 (3.2)
Bipolar II disorder	6 (6.7)	6 (9.2)	12 (7.8)
Sex, *n* (%)			
Male	23 (25.8)	20 (30.8)	43 (27.9)
Female	66 (74.2)	45 (69.2)	111 (72.1)
BMI (kg/m^2^)			
Mean (SD)	23.7 (5.29)	23.8 (4.70)	23.8 (5.03)
Onset age of illness (years)			
Mean (SD)	30.1 (11.94)	29.6 (12.04)	29.9 (11.95)
Dose used in ketamine group (*n*, %)			
0.5 mg/kg	66 (74.2)		
0.2 mg/kg	23 (25.8)		
MADRS total score (mean, SD)			
Baseline	35.1 (5.95)	37.1 (4.52)	35.9 (5.46)
Day 2	23.5 (10.66)	30.4 (9.03)	26.4 (10.54)
Day 3	24.0 (11.10)	30.4 (8.91)	26.7 (10.68)
Day 5	24.6 (11.04)	31.0 (8.48)	27.3 (10.50)
Day 7	26.2 (10.23)	30.8 (9.35)	28.1 (10.10)
Day 15	26.5 (9.85)	30.9 (9.51)	28.4 (9.93)
MSM total score			
Mean (SD)	9.2 (1.92)	9.7 (1.83)	9.4 (1.90)

Abbreviations: BMI: body mass index; MADRS: Montgomery-Åsberg Depression Rating Scale; MSM: Maudsley Staging Method; SD: standard deviation.

### Primary analysis: longitudinal trajectory of depression severity

In the primary analysis using LMM to assess the treatment effect across the 15-day observation period, the model yielded a significant main effect of treatment (Estimate = -5.27; 95% CI, -7.98 to -2.55; *P* < .001). Additionally, a significant treatment x time interaction was observed (Estimate = 0.18; 95% CI, 0.05-0.31; *p* = .006), reflecting a differential rate of change in MADRS total scores between the two groups over the follow-up interval. Baseline depression severity was a significant predictor of outcomes (*P* < .001), whereas the main effect of time was not statistically significant (*P* = .457) (Table 2).

**Table 2:** Linear mixed effect models for association between MADRS total score and ketamine treatment.^a^

Predictor	Estimate	95% CI	*P*-value
MADRS Scale Total Score			
Treatment group	−5.27	[−7.98, −2.55]	<.001^*^
Time (days)	0.04	[−0.06, 0.14]	.457
Baseline Total Score	0.89	[0.65, 1.12]	<.001^*^
Treatment group x Time Interaction	0.18	[0.05, 0.31]	.006^*^

CI: confidence interval; MADRS: Montgomery-Åsberg Depression Rating Scale

a Adjusting for age and sex

^*^Indicate the statistical significance (*P* < .05).

### Exploratory item-level analysis

The following GEE analysis was conducted as an exploratory, hypothesis-generating analysis; given the high intercorrelation among MADRS items, no correction for multiple comparisons was applied. The GEE analysis examined the odds of improvement for individual MADRS symptom domains. Patients in the ketamine group exhibited significantly higher odds of improvement on seven specific items compared to controls. Statistically significant differences were found for “Apparent sadness” (Item 1; OR = 3.46, *P* = .001), “Inner tension” (Item 3; OR = 2.01, *P* = .049), “Reduced appetite” (Item 5; OR = 5.88, *P* = .002), “Concentration difficulties” (Item 6; OR = 2.91, *P* = .025), “Lassitude” (Item 7; OR = 2.68, *P* = .030), “Pessimistic thoughts” (Item 9; OR = 3.14, *P* = .002), and “Suicidal thoughts” (Item 10; OR = 3.54, *P* = .001). Differences for “Reported sadness” (Item 2), “Reduced sleep” (Item 4), and “Inability to feel” (Item 8) did not reach statistical significance (*P* > .05) (Table 3).

**Table 3:** GEE results of primary analysis of MADRS items.^a^

Covariate	OR	95% CI	*P-*value
MADRS item 1: apparent sadness			
Treatment group	3.46	[1.62, 7.41]	.001^b^
Time	1.02	[0.97, 1.07]	.429
Baseline item score	1.56	[1.00, 2.43]	.051
Treatment group × time interaction	0.96	[0.91, 1.02]	.220
MADRS item 2: reported sadness			
Treatment group	0.26	[0.04, 1.70]	.161
Time	2.01	[0.99, 4.06]	.053
Baseline item score	0.97	[0.93, 1.02]	.237
Treatment group × time interaction	1.18	[0.64, 2.17]	.589
MADRS item 3: inner tension			
Treatment group	2.01	[1.00, 4.05]	.049^b^
Time	0.97	[0.94, 1.01]	.197
Baseline item score	1.62	[1.09, 2.40]	.017^b^
Treatment group × time interaction	1.00	[0.96, 1.05]	.880
MADRS item 4: reduced sleep			
Treatment group	1.73	[0.76, 3.93]	.193
Time	1.02	[0.96, 1.07]	.562
Baseline item score	1.31	[1.03, 1.67]	.029^b^
Treatment group × time interaction	0.93	[0.86, 1.01]	.072
MADRS item 5: reduced appetite			
Treatment group	5.88	[1.88, 18.42]	.002^b^
Time	1.11	[1.03, 1.19]	.005^b^
Baseline item score	2.29	[1.79, 2.93]	<.001^b^
Treatment group × time interaction	0.94	[0.87, 1.02]	.124
MADRS item 6: concentration difficulties			
Treatment group	2.91	[1.14, 7.40]	.025^b^
Time	1.00	[0.94, 1.06]	.918
Baseline item score	1.57	[0.86, 2.86]	.141
Treatment group × time interaction	0.98	[0.91, 1.06]	.686
MADRS item 7: lassitude			
Treatment group	2.68	[1.10, 6.55]	.030^b^
Time	1.03	[0.98, 1.08]	.224
Baseline item score	2.20	[1.46, 3.29]	<.001^b^
Treatment group × time interaction	0.96	[0.90, 1.02]	.180
MADRS item 8: inability to feel			
Treatment group	2.13	[0.81, 5.63]	.126
Time	1.00	[0.93, 1.07]	.934
Baseline item score	1.15	[0.75, 1.76]	.513
Treatment group × time interaction	1.02	[0.94, 1.11]	.590
MADRS item 9: pessimistic thoughts			
Treatment group	3.14	[1.51, 6.52]	.002^b^
Time	0.98	[0.93, 1.03]	.355
Baseline item score	1.84	[1.19, 2.83]	.006^b^
Treatment group × time interaction	1.00	[0.95, 1.07]	.882
MADRS item 10: suicidal thoughts			
Treatment group	3.54	[1.63, 7.69]	.001^b^
Time	1.03	[0.98, 1.08]	.280
Baseline item score	2.32	[1.74, 3.10]	<.001^b^
Treatment group × time interaction	0.96	[0.90, 1.02]	.178

Abbreviations: CI: confidence interval; GEE: generalized estimating equation; MADRS: Montgomery-Åsberg Depression Rating Scale; OR: odds ratio

a Adjusting for age and sex

b Indicate the statistical significance (*P* < .05).

### Focused exploratory analysis: moderation by treatment resistance

For the symptom domains that showed significant improvement in the exploratory item-level analysis, the MSM total score was further analyzed as a moderator to determine whether the treatment effect varied by the level of treatment resistance. A significant treatment × MSM interaction was identified for “Apparent sadness” (Item 1; Interaction OR = 0.58, 95% CI, 0.41-0.83, *P* = .002) and “Inner tension” (Item 3; Interaction OR = 0.67, 95% CI: 0.48-0.95, *P* = .024). The interaction terms for “Reduced appetite” (Item 5; *P* = .068), “Concentration difficulties” (Item 6; *P* = .273), “Lassitude” (Item 7; *P* = .219), “Pessimistic thoughts” (Item 9; *P* = .053), and “Suicidal thoughts” (Item 10; *P* = .083) were not statistically significant (Table 4).

**Table 4:** GEE results of exploratory analysis of MADRS items.^a^

Covariate	OR	95% CI	*P* value
MADRS item 1 apparent sadness & MSM scale total score			
Treatment group	3.89	[1.80, 8.42]	.001^b^
Time	1.01	[0.97, 1.05]	.639
Baseline item score	1.87	[1.16, 3.00]	.010^b^
MSM scale total score	1.30	[0.99, 1.70]	.058
Treatment group × time	0.97	[0.92, 1.03]	.373
Treatment group × MSM scale total score	0.58	[0.41, 0.83]	.002^b^
MADRS item 3 inner tension & MSM scale total score			
Treatment group	2.64	[1.20, 5.83]	.016^b^
Time	0.98	[0.94, 1.02]	.424
Baseline item score	1.72	[1.10, 2.69]	.017^b^
MSM scale total score	1.19	[0.90, 1.58]	.226
Treatment group × time	0.99	[0.94, 1.04]	.702
Treatment group × MSM scale total score	0.67	[0.48, 0.95]	.024^b^
MADRS item 5 reduced appetite & MSM scale total score			
Treatment group	7.42	[1.70, 32.26]	.008^b^
Time	1.10	[1.00, 1.20]	.040^b^
Baseline item score	2.81	[1.99, 3.97]	<.001^b^
MSM scale total score	1.22	[0.77, 1.92]	.390
Treatment group × time	0.94	[0.85, 1.04]	.238
Treatment group × MSM scale total score	0.59	[0.33, 1.04]	.068
MADRS item 6 concentration difficulties & MSM scale total score			
Treatment group	2.80	[1.03, 7.63]	.044^b^
Time	1.00	[0.93, 1.07]	.931
Baseline item score	1.64	[0.87, 3.09]	.130
MSM scale total score	1.09	[0.74, 1.59]	.659
Treatment group × time	0.99	[0.91, 1.08]	.848
Treatment group × MSM scale total score	0.78	[0.50, 1.22]	.273
MADRS item 7 lassitude & MSM scale total score			
Treatment group	3.32	[1.29, 8.56]	.013
Time	1.03	[0.99, 1.08]	.187
Baseline item score	2.17	[1.41, 3.33]	<.001^b^
MSM scale total score	1.11	[0.85, 1.46]	.445
Treatment group × time	0.95	[0.89, 1.01]	.088
Treatment group × MSM scale total score	0.81	[0.58, 1.13]	.219
MADRS item 9 pessimistic thoughts & MSM scale total score			
Treatment group	3.96	[1.77, 8.86]	.001^b^
Time	0.98	[0.93, 1.02]	.323
Baseline item score	2.74	[1.72, 4.35]	<.001^b^
MSM scale total score	0.98	[0.72, 1.34]	.909
Treatment group × time	1.00	[0.93, 1.06]	.902
Treatment group × MSM scale total score	0.69	[0.48, 1.01]	.053
MADRS item 10 suicidal thoughts & MSM scale total score			
Treatment group	4.47	[1.92, 10.38]	<.001^b^
Time	1.04	[0.99, 1.09]	.122
Baseline item score	3.00	[2.10, 4.28]	<.001^b^
MSM scale total score	0.93	[0.69, 1.26]	.633
Treatment group × time	0.93	[0.88, 1.00]	.037
Treatment group × MSM scale total score	0.72	[0.50, 1.04]	.083

Abbreviations: CI: confidence interval; GEE: generalized estimating equation; MADRS: Montgomery-Åsberg Depression Rating Scale; MSM: Maudsley Staging Method; OR: odds ratio

a Adjusting for age and sex

b Indicate the statistical significance (*P* < .05).

### Sensitivity analysis

To assess the robustness of the primary findings against between-trial heterogeneity, sensitivity analyses were performed by including study source^12^^,^^13^ as a covariate in both the item-level GEE models (Supplementary Table S2) and the MSM moderation GEE models (Supplementary Table S3). The treatment effects for all seven originally significant MADRS items remained statistically significant after adjustment for study source, with effect sizes highly consistent with the primary analysis (ORs ranging from 2.02 to 6.02). Similarly, the significant treatment × MSM interaction effects for Apparent Sadness (OR = 0.58, *P* = .001) and Inner Tension (OR = 0.67, *P* = .014) were preserved. These results confirm that the primary findings are not attributable to between-trial heterogeneity.

## Discussion

The present post-hoc analysis of pooled data from randomized, double-blind, controlled trials was designed to deconstruct the antidepressant efficacy of subanesthetic ketamine infusion in patients with TRD. Consistent with previous pivotal trials,^12^^,^^13^ our longitudinal analysis confirmed that a single infusion elicited a rapid and significant reduction in overall depressive symptom severity compared to control conditions over the 15-day follow-up. Beyond this global effect, item-level examination revealed that the therapeutic benefit extended across seven specific symptoms, including apparent sadness, inner tension, reduced appetite, concentration difficulties, lassitude, pessimistic thoughts, and, critically, suicidal thoughts. Most notably, our exploratory analysis further uncovered a distinct symptom-specific divergence in response patterns. We found that while the anti-suicidal efficacy of ketamine remained robust regardless of the baseline severity of treatment resistance, the therapeutic improvement in specific affective symptoms—namely apparent sadness and inner tension—was significantly moderated by the MSM scores. Specifically, the relative benefit of ketamine for these mood symptoms was attenuated in patients with higher levels of treatment refractoriness, suggesting that the resolution of apparent sadness and inner tension may be more contingent upon the underlying degree of resistance than the resolution of suicidal ideation.

Beyond the global treatment effect, our item-level analysis delineates a specific symptom profile responsive to subanesthetic ketamine. Consistent with the post-hoc analysis of the TRANSFORM-2 trial, we observed significant improvements in emotional and cognitive symptoms, specifically apparent sadness, inner tension, and concentration difficulties.^25^ However, notable divergences emerged regarding somatic and anhedonic symptoms. While previous findings highlighted “inability to feel” as a key responsive symptom, our results did not show significant improvement in this item; instead, we observed pronounced efficacy in somatic-vegetative symptoms, including reduced appetite and lassitude. This variation may reflect phenotypic differences in TRD populations, such as the prominence of somatic presentations in certain cohorts, or distinctions in the pharmacological action of racemic ketamine versus esketamine.^26^^,^^27^ Most critically, our analysis demonstrated a substantial remission of suicidal ideation, a finding that contrasts with the nonsignificant results reported previously. This discrepancy is likely attributable to distinct enrollment strategies: whereas the former trial excluded patients considered to be at serious risk for suicide, our study included patients with moderate-to-severe suicidality, thereby allowing for a more accurate characterization of ketamine’s full therapeutic spectrum.

A particularly clinically relevant finding is the efficacy of ketamine on suicidal thoughts, which was not significantly moderated by the severity of treatment resistance in our analysis. While acknowledging that the anti-suicidal effect is not entirely independent of antidepressant response^28^^,^^29^ and may vary across diagnostic subgroups,^30^ our data suggest that ketamine’s impact on suicidality is relatively resilient to the level of refractoriness compared to its effect on apparent sadness and inner tension. This finding aligns with evidence proposing that ketamine may modulate suicidality through neural pathways partially distinct from those regulating depressive mood, potentially involving the rapid normalization of glutamatergic transmission in the prefrontal cortex and anterior cingulate cortex.^31^^,^^32^ Clinically, although long-term efficacy on suicide prevention remains to be established,^33^ this finding supports the utility of ketamine as a valuable acute intervention for suicidal crises, offering a therapeutic window specifically for acute risk reduction across the spectrum of treatment resistance.

Conversely, a distinct pattern emerged regarding apparent sadness and inner tension. The significant interaction observed for apparent sadness and inner tension indicates that the therapeutic benefit of ketamine for these domains is attenuated as the severity of treatment resistance increases. Specifically, the odds of improvement diminished in patients with higher MSM scores, suggesting that the resolution of these affective symptoms is contingent upon the underlying degree of refractoriness. One speculative interpretation is that this finding may partly reflect the biological consequences of chronic, resistant depression, such as extensive synaptic loss or dendritic atrophy in mood-regulating circuits.^34^^,^^35^ While ketamine is known to promote rapid synaptogenesis,^36^ the neuroprogressive changes associated with high levels of treatment resistance may impose a ceiling on functional recovery achievable with a single infusion. Furthermore, elevated pro-inflammatory cytokines, observed in refractory cases, have been linked to a reduced antidepressant response to ketamine.^37^ However, inflammatory processes have also been implicated in suicidal ideation and behavior, suggesting that their role in the observed differential symptom response remains uncertain.^38^^,^^39^

If confirmed by prospective studies, these findings could carry implications for clinical decision-making in TRD. The observed divergence between symptom domains raises the hypothesis that treatment resistance may not constitute a uniform barrier to ketamine’s efficacy; rather, its impact may depend on the specific symptom targeted. For instance, the relative resilience of anti-suicidal efficacy across resistance levels, if replicated, would support the continued consideration of ketamine for acute suicidal crises regardless of treatment history. Conversely, the apparent attenuation of improvement in apparent sadness and inner tension at higher MSM scores suggests that future research should examine whether staging models could help identify patients who may benefit from augmentative strategies. These preliminary observations encourage further investigation into symptom-specific treatment approaches in TRD.^33^^,^^40^

Several limitations warrant consideration when interpreting these results. First, the study population consisted exclusively of Han Chinese patients from a single medical center. Given the known ethnic differences in genetic polymorphisms that may influence ketamine response,^41^ as well as potential cultural variations in symptom expression, generalization to non-Asian populations should be approached with caution. Second, the pooled control group was heterogeneous, comprising patients receiving either normal saline^12^ or midazolam at 0.045 mg/kg.^13^ Functional unblinding may have occurred in both directions: patients in the saline arm may have inferred their non-treatment assignment due to the absence of ketamine’s perceptual side effects, while patients in the ketamine arm may equally have inferred active treatment from these same effects. Furthermore, formal assessment of blinding integrity was not conducted in either contributing trial, a limitation shared with the majority of published ketamine RCTs. To evaluate whether the control group heterogeneity affected the primary findings, a sensitivity analysis adjusting for study source confirmed consistent results across all items (Supplementary Tables S2 and S3). Third, the protocol involved a single infusion with a 15-day follow-up. This design precludes conclusions regarding the durability of the observed symptom-specific divergence or the potential efficacy of repeated dosing regimens. Fourth, our cohort included mixed subanesthetic doses (0.2 and 0.5 mg/kg); while prior work within this specific population suggests dose-dependent efficacy,^12^ the sample size in this secondary analysis precluded a stratified interaction analysis by dose level. Finally, regarding psychometric properties, single-item analyses may possess lower reliability compared to the aggregate total score.^42^ However, our use of the GEE model with a binary outcome framework was specifically adopted to mitigate the distributional instability inherent in analyzing individual item scores. Additionally, apparent sadness in the standard MADRS is rated based on the clinician’s direct observation of the patient’s speech, facial expression, and posture. As such, this item captures outward affective expression, which may not fully correspond to the patient’s internal emotional state. This psychometric consideration should be taken into account when interpreting the clinical significance of the observed interaction effects.

In conclusion, this study offers an exploratory examination of the therapeutic effects of subanesthetic ketamine in treatment-resistant depression, suggesting potential differences across symptom domains. While ketamine was associated with reductions in suicidal ideation across levels of treatment resistance, improvements in apparent sadness and inner tension appeared more limited among patients with higher levels of refractoriness. These observations may point to heterogeneity in treatment response across symptom domains; however, given the exploratory nature of the analysis, these findings should be interpreted cautiously. Further studies are needed to determine whether staging models, such as the MSM, may help clarify patterns of response and inform treatment strategies in treatment-resistant depression.

## Supplementary Material

Supplementary_material_R1_pyag021

## Data Availability

The data that support the findings of this study are available on request from the corresponding author. The data are not publicly available due to the ethical regulation in Taiwan.
