# Supplementary material for: Distinct therapeutic profiles of ketamine in treatment-resistant depression: an exploratory analysis
Source: Int J Neuropsychopharmacol. 2026 Apr 30;29(5):pyag021. doi: 10.1093/ijnp/pyag021 (PMC13192412; doi:10.1093/ijnp/pyag021)
Supplement: Supplementary_material_R1_pyag021 [file supplementary_material_r1_pyag021.docx]

**Supplementary material**

**Table S1. Characteristics of the Three Pooled Clinical Trials**

| Trial | Su et al. (2017) | Su et al. (2023) | Chen et al. (2019) |
| --- | --- | --- | --- |
| Design | Double-blind RCT | Double-blind RCT | Open-label ketamine +  double-blind DCS maintenance RCT |
| N enrolled | 71 | 84 | 32 |
| N in current analysis | 71 (100%) | 83 (99%) | 0 (0%) |
| Ketamine dose | 0.5 mg/kg or  0.2 mg/kg IV | 0.5 mg/kg IV | 0.5 mg/kg IV |
| Control condition | Normal saline | Midazolam 0.045 mg/kg IV | D-cycloserine vs. oral placebo |
| Follow-up time points | baseline, D2, D3, D5, D7, D15 | baseline, D2, D3, D5,  D7, D15 | Weekly for 6 weeks |
| Blinding | Double-blind | Double-blind | Open-label (infusion phase) |
| Reference | Ref. 8 | Ref. 9 | Ref. 16 |

Note: Chen et al. (2019) contributed no participants to the current analysis, as its outcome assessment schedule did not include a post-infusion Day-15 MADRS assessment.

**Table S2. Sensitivity Analysis^a^: Item-Level GEE with Study Source Covariate (Treatment Effect)**

| MADRS Item | OR | 95% CI | P value |
| --- | --- | --- | --- |
| 1 – Apparent sadness | 3.61 | [1.70, 7.65] | 0.001^*^ |
| 2 – Reported sadness | 2.07 | [1.02, 4.21] | 0.045^*^ |
| 3 – Inner tension | 2.02 | [1.00, 4.10] | 0.049^*^ |
| 4 – Reduced sleep | 1.73 | [0.74, 4.06] | 0.204 |
| 5 – Reduced appetite | 6.02 | [1.97, 18.43] | 0.002^*^ |
| 6 – Concentration difficulties | 2.75 | [1.06, 7.14] | 0.037^*^ |
| 7 – Lassitude | 2.60 | [1.04, 6.47] | 0.041^*^ |
| 8 – Inability to feel | 2.16 | [0.83, 5.66] | 0.117 |
| 9 – Pessimistic thoughts | 3.02 | [1.45, 6.27] | 0.003^*^ |
| 10 – Suicidal thoughts | 3.37 | [1.53, 7.39] | 0.002^*^ |

CI: confidence interval; GEE: generalized estimating equations; MADRS: Montgomery-Åsberg Depression Rating Scale; OR: odds ratio

^a^ Adjusting for age, sex, and study source

^*^ indicate the statistical significance (p < 0.05)

**Table S3. Sensitivity Analysis^a^: MSM Moderation GEE with Study Source Covariate (Treatment × MSM Interaction)**

| MADRS Item | Interaction OR | 95% CI | P value |
| --- | --- | --- | --- |
| 1 – Apparent sadness | 0.58 | [0.41, 0.80] | 0.001^*^ |
| 3 – Inner tension | 0.67 | [0.48, 0.92] | 0.014^*^ |
| 5 – Reduced appetite | 0.65 | [0.41, 1.04] | 0.072 |
| 6 – Concentration difficulties | 0.83 | [0.56, 1.24] | 0.371 |
| 7 – Lassitude | 0.80 | [0.58, 1.09] | 0.159 |
| 9 – Pessimistic thoughts | 0.70 | [0.50, 0.97] | 0.035^*^ |
| 10 – Suicidal thoughts | 0.73 | [0.52, 1.03] | 0.069 |

CI: confidence interval; GEE: generalized estimating equations; MADRS: Montgomery-Åsberg Depression Rating Scale; MSM: Maudsley Staging Method; OR: odds ratio

^a^ Adjusting for age, sex, and study source

^*^ indicate the statistical significance (p < 0.05)
